# Improvement of systemic sclerosis-associated digital ulcers after ultraviolet A1 phototherapy

**DOI:** 10.1016/j.jdcr.2023.11.005

**Published:** 2023-11-30

**Authors:** Meagan M. Olivet, Kevin Yang, Lauren V. Graham

**Affiliations:** a UAB Heersink School of Medicine, Birmingham, Alabama; b Department of Dermatology, University of Alabama at Birmingham, Birmingham, Alabama

**Keywords:** digital ulcers, phototherapy, scleroderma, systemic sclerosis, ultraviolet radiation, UVA1

## Introduction

Systemic sclerosis is a connective tissue disorder characterized by excessive collagen production and deposition of collagen both in the skin and internal organs, which leads to vascular complications and fibrosis. Digital ulcers represent a significant complication of systemic sclerosis that severely impair a patient’s ability to use their hands and may lead to infection and amputation.

## Case report

A 47-year-old female with a history of systemic sclerosis managed by rheumatology presented to dermatology clinic in 2012 with worsening stiffness and ulcerations of her fingers of 2-year duration. At that time, she was taking hydroxychloroquine, azathioprine, and prednisone. Her examination was notable for smooth, taut skin of the hands with multiple ulcers on the bilateral finger pads, but she was subsequently lost to follow-up.

**Fig 1:**
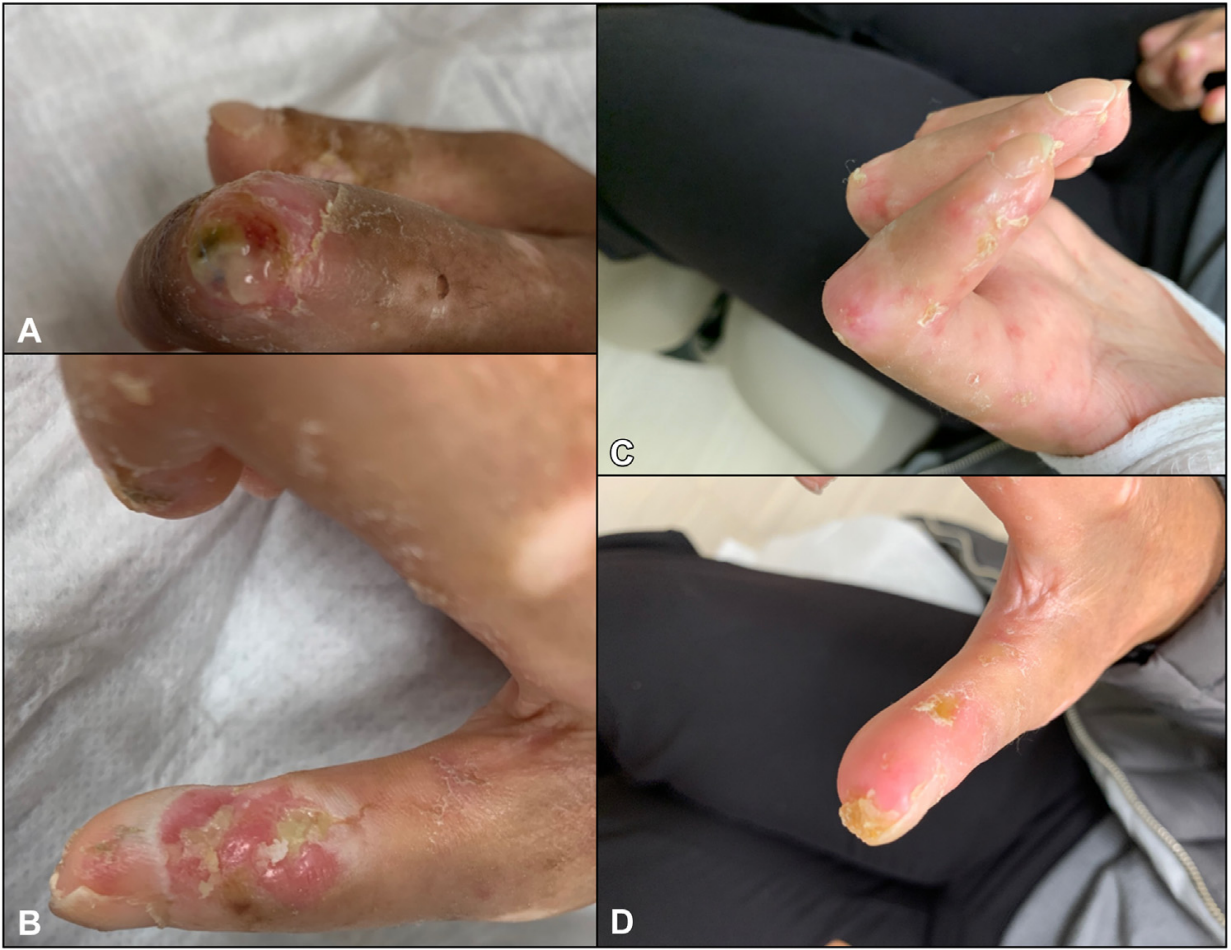
Digital ulcers of second digit **(A)** and thumb **(B)** before initiation of UVA1 phototherapy; Healing ulcerations noted on second digit **(C)** and thumb **(D)** after 20 sessions of UVA1 phototherapy **(C, D)**.

She reestablished with dermatology in July 2021, at which time she was maintained on sildenafil, pentoxifylline, and nitroglycerin ointment to the fingers with little benefit. In addition, she was counseled on using mupirocin 2% ointment to ulcers and starting vinegar soaks to prevent infection. Her systemic regimen at this point consisted of hydroxychloroquine 200 mg twice daily, mycophenolic acid 360 mg 3 times daily, prednisone 5 mg 3 times daily, and intravenous immunoglobulin 2 g/kg every 4 weeks. She continued to see no improvement in her digital ulcers with development of new ulcers (Fig 1). Given the lack of improvement despite multiple systemic and topical treatments, she was started on UVA1 (hand/foot, Daavlin SL3000) phototherapy 3 times a week.

At follow-up, she noted improvement in her ulcers with improvement in healing time, after having undergone 20 sessions, reaching a max dose of 50 J/cm^2^ (Fig 1). She reported continued ulceration of the fingertips which were notably untreated due to phototherapy to the dorsal surfaces of the hands unable to reach the tips of her contracted fingers. She also had improvement in skin tightness of the forearms over which she received phototherapy. Given these positive changes, she was subsequently started on UVA1 to the palmar surface of the hands to include exposure to digital ulcers on her contracted fingertips with similar beneficial effect.

## Discussion

The pathogenesis of digital ulcers in systemic sclerosis is complex and continues to be elucidated. Ischemia is thought to play a key role in their development, particularly at the fingertips, whereas digital ulcers located in extensor areas of the hands may be driven more by recurrent microtrauma.^1^ Additionally, increased prevalence of macrovascular disease may be implicated with increased formation of arteriovenous anastomoses.^2^ Calcinosis may also instigate a local inflammatory response that can lead to digital ulcer formation. Ultraviolet A1 (UVA1) phototherapy can be a valuable therapy to mitigate inflammation and reduce dysfunctional vascularity at the root of digital ulcers in systemic sclerosis.

Although there is robust literature on the benefits of UVA1 on morphea and, to a lesser extent, on systemic sclerosis,^3^ limited literature exists on the efficacy of UVA1 in the treatment of digital ulcers. In a case report by Inoue et al,^4^ a patient was treated with a course of oral psoralen plus UVA therapy; following therapy, there was noted improvement in the tension of the skin and in the digital ulcers. Biopsies that were taken before and after treatment demonstrated loosening of the collagen bundles and immunofluorescence showed increased numbers of CD34+ cells.^4^ This underlines the efficacy in reducing sclerotic changes and edema. Additionally, in a case study of patients with Raynaud phenomenon secondary to systemic sclerosis or systemic lupus erythematosus, there was improvement of digital ulcers noted in all 11 cases with 75% of patients having complete healing.^5^

Although incompletely understood, the major mechanisms of UVA1 are thought to include induction of apoptosis, cytokine production, and fibroblast activity.^6^, ^7^, ^8^, ^9^ From a standpoint of reducing inflammation that may lead to improvement of digital ulcers, UVA1 suppresses proinflammatory cytokines, including tumor necrosis factor alpha, interleukin-12, and interferon gamma.^7^^,^^8^

In addition to these effects, UVA1 has also been reported to modify endothelial regulation and transformation. In a study of cultured human fibroblasts, UVA1 exposure was found to increase vascular endothelial growth factor expression.^10^ Similarly, biopsies taken from skin of patients with systemic sclerosis demonstrated increased CD34 and vascular endothelial growth factor expression after UVA1 phototherapy, suggesting angiogenesis.^4^ By inducing neovascularization of sclerotic skin, UVA1 may help to correct the vascular processes underlying the pathogenesis of digital ulcers.

Although the efficacy of UVA1 therapy shows imminent promise for patients, there are limitations to consider. This treatment requires a high level of commitment from the patient given that it can be time consuming and challenging for patients to regularly attend clinic visits. Furthermore, UVA1 therapy can be painful, especially when up-titrating is required. Financial barriers may also be considered for patients with high insurance copays for procedures. Considering a patient’s social determinants of health, ability to regularly present to clinic, and overall tolerability of the treatment are key when considering UVA1 therapy.

Reducing the burden of disease for systemic sclerosis patients with digital ulcers is crucial for improving patients’ quality of life. Previous therapies have been more supportive and strived to decrease complications, such as infection. Utilizing UVA1 therapy, however, can improve quality of life for patients by reducing the burden of digital ulcers and combating vasculopathy in systemic sclerosis patients.

## Conflicts of interest

None disclosed.
